# Ethics and Jurisprudence

**Published:** 1885-09-20

**Authors:** 


ETHICS AND JURISPRUDENCE.--The Society of Medical Jurisprudence and State Medicine was organized in New York, 1883. At their last meeting, in May, a very interesting address was delivered by Hon. W. H. H. Russell upon the subject of Professional Ethics. He takes high ground, and advocates a code of ethics even among lawyers. He remarks: "If every lawyer and disciple of the great Blackstone, Story, and Kent were to exemplify the true principles of Law, Equity, and Justice, what a magnificent system of Jurisprudence we would have in this country! Government, society, and religion would then triumise and glow more beautiful than the prospective electric light which is to gleam and glitter from that noble gift of warm-hearted France, which is soon to ornament our harbor with the ideal statue of 'Liberty enlightening the World!'"

